# Supplementary material for: Quantitatively defining species boundaries with more efficiency and more biological realism
Source: Commun Biol. 2022 Jul 28;5:755. doi: 10.1038/s42003-022-03723-z (PMC9334598; doi:10.1038/s42003-022-03723-z)
Supplement: Supplementary file 1 — Supplementary Information [file 42003_2022_3723_MOESM1_ESM.pdf]

Quantitatively defining species boundaries with more efficiency and more biological realism

Jordan Douglas[1,*] and Remco Bouckaert[1]

[1]School of Computer Science, The University of Auckland, Auckland, New Zealand
[*]jordan.douglas@auckland.ac.nz.

June 21, 2022

Supplementary Information

Our two well-calibrated simulation studies are presented in Fig. S1 and S2 below. The coverage of each parameter is close to 95% thus providing confidence in the validity of these methods.

We also explored the effect of varying ϵ values at doing inference on simulated data, where the true value of ϵ was known (Fig. S3). Species trees were simulated such that their heights were quite small (95% credible interval 0.00009 – 0.07 substitutions per site) – reflecting the use case where taxa are closely related and therefore species delimitation can be a difficult and blurred problem. Note that these are the same 100 simulated datasets presented in Fig. S2.

When the true value of ϵ was used during inference (i.e. $\epsilon = 10^{-4}$), the posterior cluster supports were accurate. When the incorrect value of ϵ was used ($\epsilon = 10^{-6}, 10^{-5}, 10^{-3}$), the cluster posterior supports were slightly overconfident (positive bias) or underconfident (negative bias) but they were still reasonably accurate, suggesting a moderate degree of robustness. However, the largest threshold considered $\epsilon = 10^{-2}$ gave nonsensical results and could not estimate cluster support. This is an unsurprising result (given that many simulated trees here had a root height less than 10^{-2} substitutions per site), and is congruent with our user guidelines for selecting ϵ described in the main article (where we suggest that $\epsilon = 10^{-2}$ is too large for biological datasets). We also found little bias in the inferred number of clusters for $\epsilon = 10^{-6}, 10^{-5}, 10^{-4}$, but the inferred number of clusters started to decline for $\epsilon = 10^{-3}$, and even further for $\epsilon = 10^{-2}$ (bottom right panel of Fig. S3).

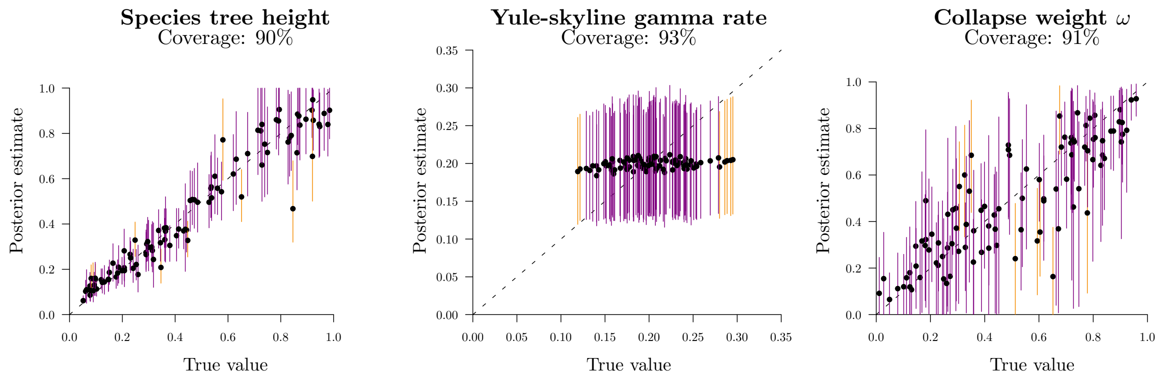

Fig. S 1: Well-calibrated simulation study of the YSC model under a multispecies coalescent framework, with gene trees integrated out (SNAPPER). The species tree consisted of 40 taxa, and four samples were assigned to each species tree taxon *a priori*. Each of the 100 gene trees are associated with one SNP. The mean parameter estimates (under the posterior distribution) are indicated by black circles, and 95% highest posterior density (HPD) intervals are coloured dark if the true value is in the interval, or light otherwise. Most of the terms have close to 95% coverage under the 95% HPD, therefore suggesting that the model is both valid and correctly implemented. Omitted from this figure are the coalescent rates; which also have close to 95% coverage. “True” values were sampled from the joint prior distribution using MCMC.

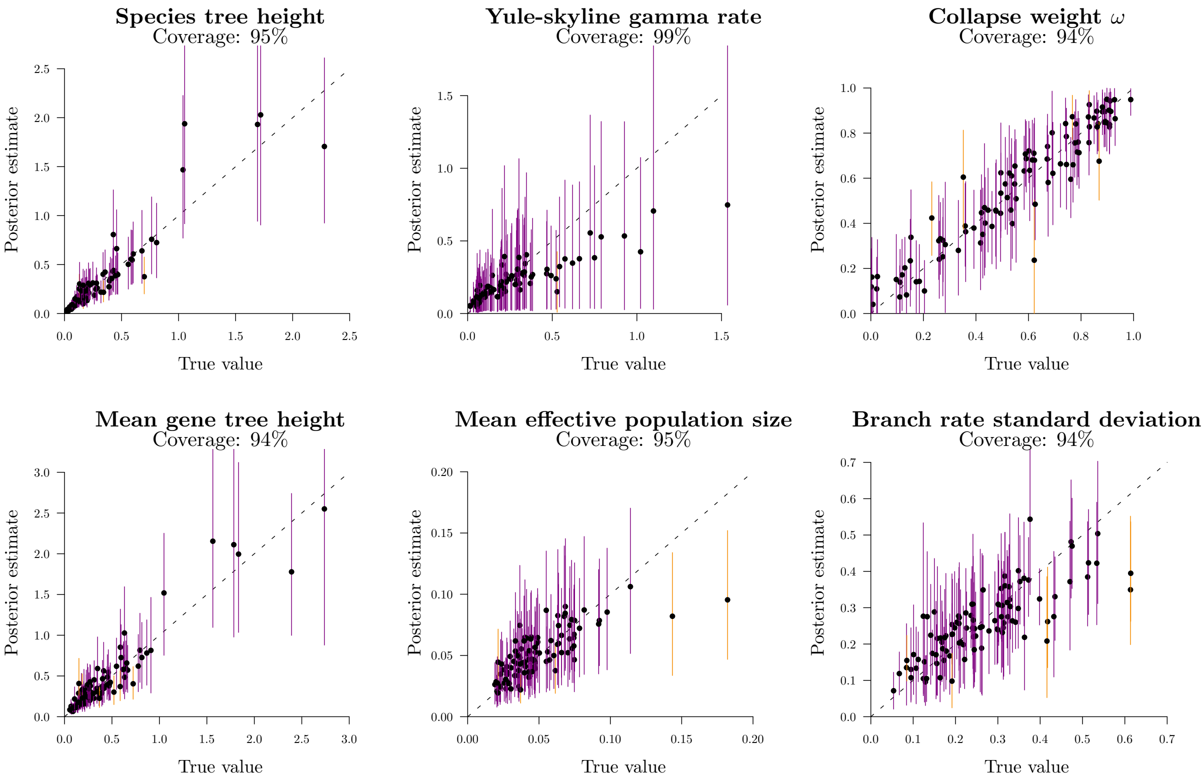

Fig. S 2: Well-calibrated simulation study of the YSC model under a multispecies coalescent framework, with gene trees estimated (StarBeast3). The species tree consisted of 40 taxa, and one sample was assigned to each *a priori*. Each gene tree was inferred from a respective nucleotide sequence 0.5kb in length. Mean gene tree heights were averaged across 4 gene trees, and the multispecies relaxed clock model was applied (Ogilvie et al., 2017). These results suggest that the model is likely to be both valid and correctly implemented (see Fig. S 1 for figure notation). Omitted from this figure are the gene-tree HKY substitution model parameters; which also have close to 95% coverage. Parameters were directly sampled from the prior in order to capture the generative process.

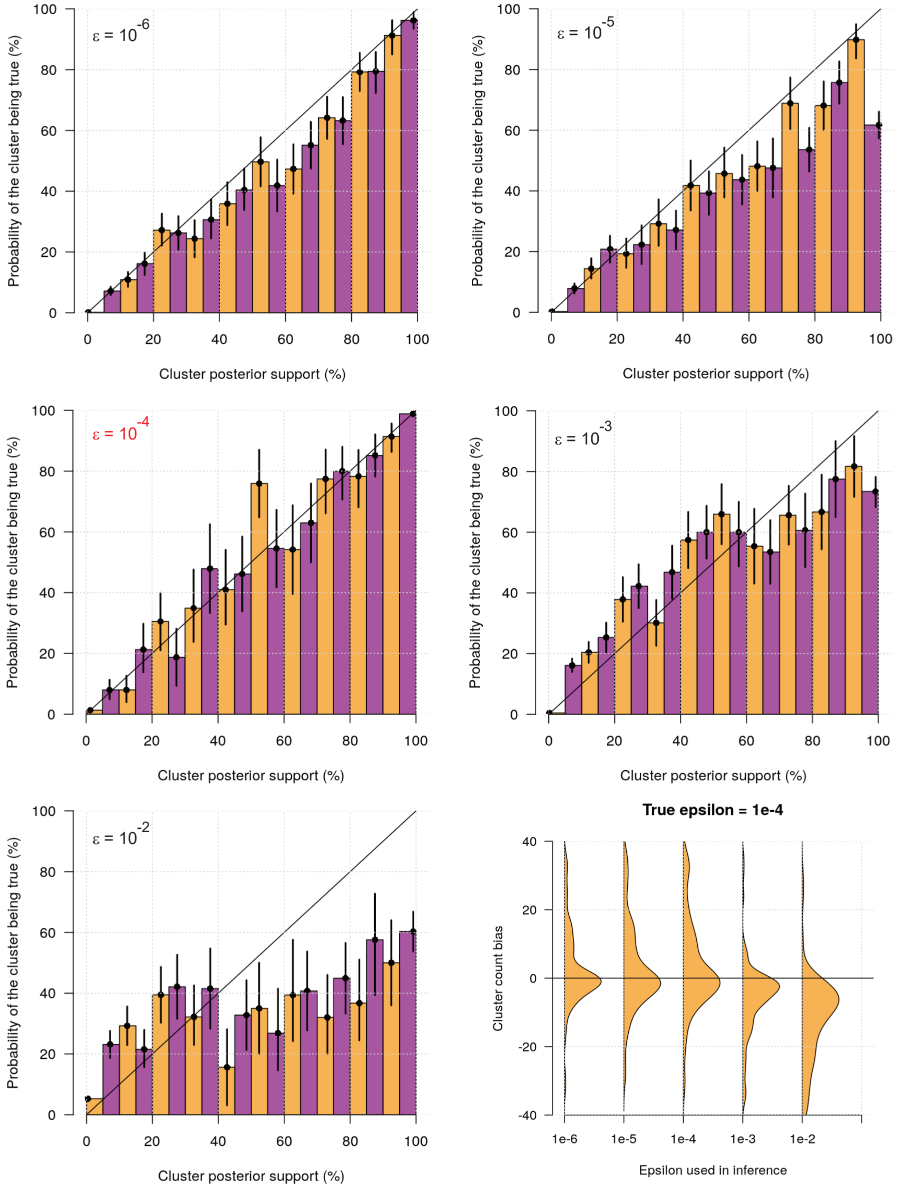

Fig. S 3: Sensitivity of species delimitation to ϵ. First, we simulated 100 datasets using StarBeast3, each with 40 taxa and 16 genes trees, where $\epsilon = 10^{-4}$. Then, we inferred the clustering using varying values of $\epsilon = 10^{-6}, 10^{-5}, 10^{-4}, 10^{-3}, 10^{-2}$, and compared the posterior cluster support (x-axis) with the probability of the cluster existing (y-axis). This is the same experiment described at the top of Figure 2 of the main article, with varying thresholds. Bottom right: we compared the maximum *a posteriori* cluster count estimate with the true count, and plotted the distribution across 100 datasets. The bias is equal to the true number of clusters minus the inferred number, and has a domain of (-40, 40) because there are 40 taxa in the species tree.
